# Infectious Keratitis Management: 10-Year Update

**DOI:** 10.3390/jcm14175987

**Published:** 2025-08-25

**Authors:** Neel D. Pasricha, Pablo Larco, Darlene Miller, Diego S. Altamirano, Jennifer R. Rose-Nussbaumer, Eduardo C. Alfonso, Guillermo Amescua

**Affiliations:** 1 Department of Ophthalmology, University of California San Francisco, San Francisco, CA 94158, USA; 2 Francis I. Proctor Foundation, University of California San Francisco, San Francisco, CA 94158, USA; 3 Bascom Palmer Eye Institute, University of Miami, Miami, FL 33136, USA; 4 Instituto Oftalmológico Integral, Santiago 8320214, Chile; 5 Department of Ophthalmology, Stanford University, Palo Alto, CA 94305, USA

**Keywords:** corneal ulcer, infectious keratitis, antimicrobial therapy

## Abstract

Infectious keratitis (IK), including bacterial, fungal, parasitic, and viral etiologies, continues to represent a significant cause of ocular morbidity in the United States and around the world. Corneal scraping for smears and cultures remains the gold standard in diagnosing IK; however, molecular diagnoses, including metagenomic deep sequencing (MDS), are promising emerging diagnostic tools. Despite recent interest in procedural treatment such as riboflavin photoactivated chromophore corneal collagen cross-linking (PACK-CXL) and Rose Bengal photodynamic antimicrobial therapy (RB-PDAT), medical treatment advances have remained stagnant. **Methods**: This review highlights IK pathogens obtained from corneal cultures at Bascom Palmer Eye Institute (BPEI) from 2011 to 2021 and provides the current BPEI algorithms for initial management of IK or as a referred clinically worsening patient. The roles of corticosteroid therapy, PACK-CXL, and RB-PDAT for IK are also summarized. **Results**: A total of 9326 corneal cultures were performed at BPEI between 2011 and 2021, and only 3609 (38.7%) had a positive organism identified, of which bacteria were the most common (83.4%). Fortified vancomycin and tobramycin are recommended as first-line medical therapy for IK patients based on culture sensitivity data for the top Gram-negative (*Pseudomonas aeruginosa*) and Gram-positive (*Staphylococcus aureus*) bacteria. PACK-CXL and RB-PDAT may benefit IK patients with corneal melting and fungal IK, respectively. **Conclusions**: Drug holidays, minimizing contamination, and optimizing sample order are crucial to maximizing corneal culture positivity. PACK-CXL and RB-PDAT are promising procedural advancements for IK therapy.

## 1. Introduction

Infectious keratitis (IK) remains a large unmet clinical need worldwide and the fifth leading cause of global blindness [1,2]. One major risk factor for IK in the USA is contact lens use, with an incidence of IK in 130 per 100,000 contact lens wearers compared to only 14 per 100,000 in non-contact lens wearers [3]. Other risk factors for IK include ocular trauma, ocular surface disease, lid disease, and prior ocular surgery [4,5,6]. In 2010 in the USA, approximately 1 million IK clinic visits occurred, accounting for direct healthcare expenditure costs of USD 135 million annually [5,7]. More recently, an IRIS^®^ Registry study showed that IK may account for 6.5% of all ophthalmology visits in the USA [8]. An estimated 30% of IK patients ultimately endure at least moderate vision loss, defined as ≤20/60 best-corrected visual acuity, in their affected eye [9]. Thus, the prompt identification and treatment of IK is imperative to prevent morbidity.

There have been no new Food and Drug Administration (FDA)-approved topical ophthalmic antibiotics since the fourth-generation fluoroquinolone, besifloxacin 0.6% (Besivance; Bausch + Lomb, Laval, QC, Canada), in 2009. There have been diagnostic and procedural advancements in IK management over the past decade. Metagenomic deep sequencing (MDS) allows for hypothesis-free probing of all DNA and RNA in a sample, including parasites and viruses, providing improved diagnostic sensitivity and accuracy [10,11]. Adjuvant riboflavin photoactivated chromophore corneal collagen cross-linking (PACK-CXL) has some benefit in bacterial IK but limited efficacy in fungal IK [12,13,14]. Rose Bengal photodynamic antimicrobial therapy (RB-PDAT) may have improved adjuvant clinical utility over PACK-CXL, especially for fungal IK [15,16,17,18].

Bascom Palmer Eye Institute (BPEI) serves as a tertiary referral center that cares for more than 1000 patients with IK annually. We have previously published algorithms for managing IK in 2012 [19]. Between 2011 and 2021, 9326 corneal cultures were performed at BPEI. This review provides a summary of corneal culture results and revised IK algorithms from BPEI over the past decade. Additionally, a comprehensive update on IK management, including the roles of topical corticosteroids, PACK-CXL, and RB-PDAT, is summarized.

## 2. Materials and Methods

All corneal cultures received by the Ocular Microbiology Laboratory at BPEI between 2011 and 2021 were reviewed. Our previously published algorithms for managing IK were revised [19]. A literature search for human clinical studies on PACK-CXL and RB-PDAT for IK was performed.

## 3. Results

### 3.1. Corneal Culture Results

**Figure 1:**
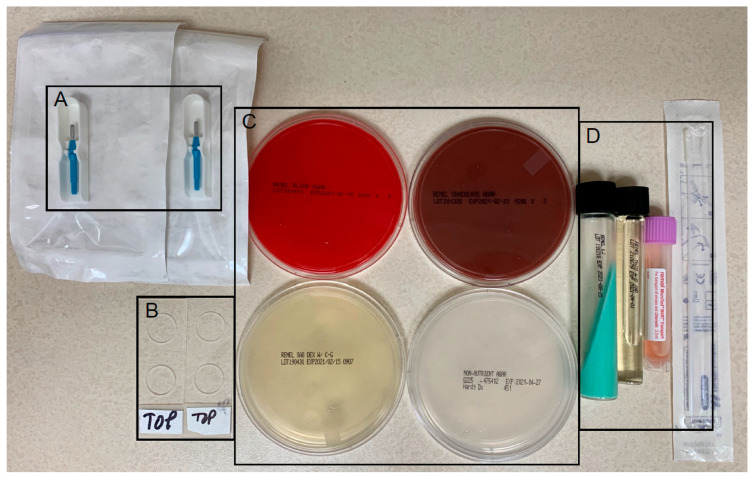
Corneal culture supplies. (**A**) #69 mini-blades for corneal scrapings. (**B**) Two microbiology slides, one for Gram stain (bacteria) and another for potassium hydroxide (KOH) stain (fungi). (**C**) Blood agar (top left, most bacteria), chocolate agar (top right, most bacteria, including fastidious respiratory bacteria, and most fungi), potato flake agar (bottom left, fungi), and non-nutrient agar (bottom right, acanthamoeba) plates. (**D**) Löwenstein–Jensen medium (left, mycobacteria), thioglycolate broth (middle, aerobic versus anaerobic microbes), and universal transport medium with flocked swab (right, viruses and chlamydia).

The BPEI corneal culture supplies are shown in Figure 1. Of the 9326 corneal cultures performed at BPEI between 2011 and 2021, only 3609 (38.7%) had a positive organism identified. This low culture-positive rate is consistent with our previously reported rate from 2010 (280/736, 38%) [19]. Over 90% of IK patients in our series were pretreated with topical antimicrobial therapy prior to obtaining corneal cultures, the likely etiology of the low culture-positive rate. Table 1 summarizes the organisms identified by corneal culture. Bacteria were the most common (83.4%), with Gram-negative more common than Gram-positive (46.9% vs. 34.1%). *Pseudomonas aeruginosa* was the top Gram-negative bacterium identified and was found to have 100% sensitivity to tobramycin, consistent with a recently published retrospective cohort study from the University of Pittsburgh [20]. *Staphylococcus aureus* was the top Gram-positive bacterium identified and was found to have 96% sensitivity to vancomycin, 92% to trimethoprim/polymyxin B, and 61% to moxifloxacin. For fungal IK (13.4% of IK patients), mold was more common than yeast (10.7% vs. 2.7%). We recommend natamycin for molds, such as *Fusarium*, and amphotericin B or voriconazole for yeasts, such as *Candida* and *Aspergillus*. Oral azoles, including voriconazole and posaconazole, can be helpful adjuvant therapy for fungal IK but require weekly monitoring of liver function due to risk of hepatotoxicity, and ophthalmologists should warn patients about the possible side effect of hallucinations.

### 3.2. The First Algorithm: New IK Patient

**Figure 2:**
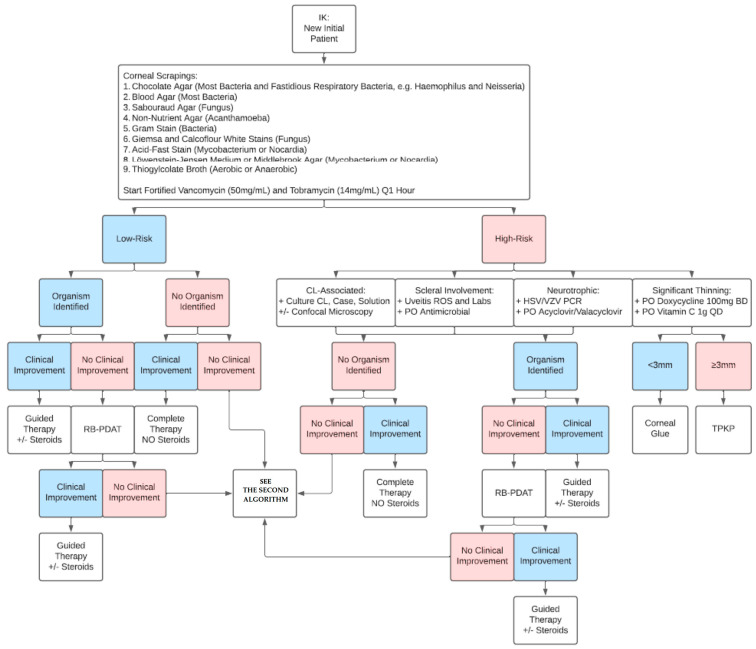
Algorithm for a new infectious keratitis (IK) patient. CL = contact lens; HSV/VZV = herpes simplex virus/varicella zoster virus; RB-PDAT = Rose Bengal photodynamic antimicrobial therapy; ROS = review of systems; TPKP = therapeutic penetrating keratoplasty.

Figure 2 shows the BPEI algorithm for approaching a new IK patient. Based on our experience, corneal cultures are recommended for all cases of IK, regardless of disease stage. For patients with high-risk IK features, including contact lens wear, scleral involvement, neurotrophic keratopathy, and corneal thinning, we recommend additional measures. In contact-lens-wearing IK patients, the contact lens, case, and solution should be obtained and cultured. The contact lens should be placed on the chocolate agar plate for the microbiologist to mince. In cases of scleral involvement, a uveitis review of systems and lab workup is warranted. This includes infectious causes for scleritis, including tuberculosis (PPD/QuantiFERON-TB Gold), syphilis (RPR/FTA-ABS), and Lyme disease, and non-infectious causes for scleritis, including rheumatoid arthritis (ANA, RF, anti-CCP), granulomatosis with polyangiitis (ANCA), sarcoidosis (chest X-ray, ACE, lysozyme), and non-specific inflammation (CBC, ESR, CRP). Corneal sensation should be checked in all new IK patients prior to instilling any eye anesthetic drops in the clinic. If the patient is neurotrophic, we consider checking HSV/VZV PCR and starting the patient on treatment dose oral valacyclovir (1 g three times a day, renally adjusted if needed). For patients with corneal thinning from active tissue melting, oral doxycycline and vitamin C are used to suppress corneal collagenase and matrix metalloproteinase (MMP) activity. We have a low threshold to use cyanoacrylate glue given its globe-stabilizing and antimicrobial properties [21]. If therapeutic penetrating keratoplasty (TPK) is needed, the host corneal tissue should be sent for both microbiology (on chocolate agar plate) and pathology. To avoid the need for large-diameter TPKs and fresh donor corneal tissue, corneal patch grafting using irradiated corneal tissue can be a viable option, especially for peripheral IK [22]. Similarly, conjunctival pedicle flaps may be considered for peripheral IK, such as cataract wound infection [23]. In cases where no organism is identified, we do not recommend the addition of topical corticosteroids, given concern for atypical organisms like fungus, nocardia, or acanthamoeba. In cases of non-nocardia bacterial IK, however, we do recommend adding topical corticosteroids [24]. If the organism is identified but the patient is clinically worsening, RB-PDAT is considered.

### 3.3. The Second Algorithm: Referred Clinically Worsening IK Patient

**Figure 3:**
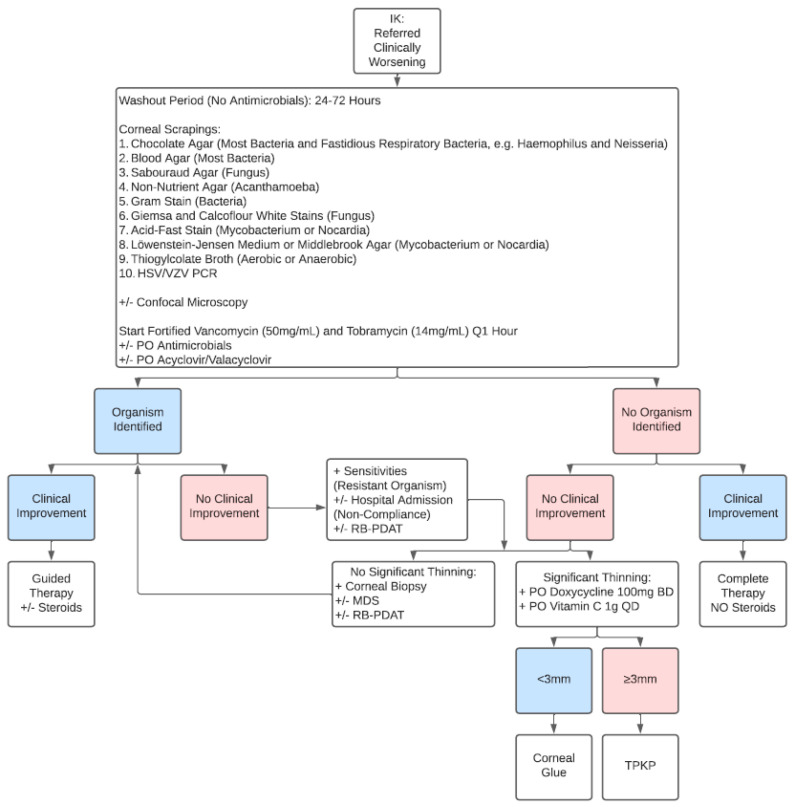
Algorithm for a referred clinically worsening infectious keratitis (IK) patient. HSV/VZV = herpes simplex virus/varicella zoster virus; MDS = metagenomic deep sequencing; RB-PDAT = Rose Bengal photodynamic antimicrobial therapy; TPKP = therapeutic penetrating keratoplasty.

Figure 3 shows the BPEI algorithm for approaching a referred clinically worsening IK patient. Since these patients are usually already on topical antimicrobial therapy, we recommend a washout period of 24–72 h with no antimicrobials to increase corneal culture yield as long as there is no severe corneal melting or impending corneal perforation [19]. Confocal microscopy is recommended for these IK patients to help identify atypical organisms like fungus or acanthamoeba [25]. Although confocal microscopy offers a rapid and noninvasive method of IK diagnosis with excellent sensitivity and specificity in the hands of skilled users, its high cost, limited accessibility, and user dependability present challenges [26]. In cases where an organism is identified but there is no clinical improvement, the provider must check sensitives for resistance and query poor patient compliance as a potential issue, with a low threshold for hospital administration. If the patient continues to worsen but there is no significant corneal thinning, a corneal biopsy and molecular diagnoses, such as PCR or MDS, can be useful to identify atypical organisms. For corneal biopsy, if there is anterior corneal pathology, our preferred technique is to use a 3 mm dermatologic trephine and crescent blade to excise an anterior lamellar corneal button encompassing the area of corneal ulceration, along with an edge of non-infected cornea, if possible. The corneal button is sent for both microbiology (on chocolate agar plate) and pathology analyses. If there is posterior corneal pathology, a 6-0 silk suture pass is performed, entering from the non-infected cornea and exiting through the infected cornea, then sent for microbiology (on chocolate agar plate) analysis only, since pathology analysis is not possible [27].

### 3.4. Corticosteroid Therapy

Table 2 summarizes recommendations for corticosteroid therapy in IK patients. For non-nocardia and non-mycobacteria bacterial IK, we recommend initiation of topical corticosteroids within 48 h. In patients with acanthamoeba IK, corticosteroid therapy is more controversial and should only be used in cases of severe inflammation or scleritis after appropriate antiamoebic therapy. We recommend a combination of biguanide (e.g., chlorhexidine 0.02%) and diamidine (propamidine isethionate 0.1%) as first-line therapy for acanthamoeba IK [28]. If progressing, RP-PDAT and oral miltefosine therapy are considered. These patients can subsequently develop severe stromal inflammation that can lead to corneal melting; therefore, we recommend simultaneous treatment with oral and/or topical corticosteroid [29,30]. The Parasitic Ulcer Treatment Trial (PUTT) is a multicenter, parallel-group, RCT to determine whether including topical corticosteroids in a regimen for acanthamoeba IK will improve vision and should complete enrollment in 2028 (ClinicalTrials.gov Identifier: NCT06213649). For viral IK, topical corticosteroids are used in conjunction with oral antiviral therapy for stromal and endothelial involvement but contraindicated in the initial management of epithelial involvement. As mentioned previously, we do not recommend corticosteroid therapy in cases of unknown IK, given the risk of atypical organisms like fungus, nocardia, mycobacteria, or acanthamoeba. Steroid-sparing immunotherapy, including topical cyclosporine and tacrolimus, may be beneficial in patients when topical corticosteroids are contraindicated, such as in fungal keratitis [31,32].

### 3.5. PACK-CXL

PACK-CXL combines topical riboflavin and UV-A light to limit microbial replication, damage pathogen cell walls, and increase corneal stromal resistance to enzymatic degradation. The first RCT for PACK-CXL failed to demonstrate a statistically significant difference in time to corneal healing between adjunctive PACK-CXL and non-CXL groups in patients with advanced IK and coexisting corneal melting. However, adjunctive PACK-CXL was found to halt corneal melting and prevent corneal perforation in 100% of treated patients compared to 84% of non-CXL patients [39]. Table 3 summarizes the recent clinical studies evaluating PACK-CXL. Of note, multiple clinical studies have demonstrated poor efficacy of PACK-CXL in cases of fungal IK [13,14]. Results for bacterial IK are mixed, however, with one RCT showing an 89% success rate in first-line standalone PACK-CXL and another RCT showing no benefit of adjuvant PACK-CXL in addition to standard-of-care topical moxifloxacin 0.5% [40,41]. The Steroids and Cross-linking for Ulcer Treatment Trial (SCUT II) is an international, randomized, sham and placebo-controlled, three-arm clinical trial that began patient recruitment in September 2020 and is expected to complete by 2024 that will randomize patients with smear-positive bacterial corneal ulcers into one of three treatment arms: (1) Topical Moxifloxacin 0.5% with topical placebo and sham CXL; (2) Topical Moxifloxacin 0.5% plus topical Difluprednate 0.05% with sham CXL; or (3) CXL plus topical Moxifloxacin 0.5% plus topical Difluprednate 0.05% [42]. Of note, SCUT II will perform CXL with a UV fluence of 5.4 J/cm^2^, which has demonstrated lower antibacterial efficacy than higher UV fluences [43].

### 3.6. RB-PDAT

RB-PDAT combines topical Rose Bengal dye and green light to generate reactive oxygen species (ROS). Table 4 summarizes the clinical studies evaluating RB-PDAT. Given the limited efficacy of PACK-CXL in cases of fungal IK, RB-PDAT was initially tested as adjuvant therapy in fungal IK patients with promising in vitro results [15]. A recent case series in India demonstrated excellent efficacy of adjuvant RB-PDAT in *Fusarium* IK but poor response in *Aspergillus* and *Acremonium* IK [17]. In the most recent and largest clinical study of RB-PDAT at our institution, we found that adjuvant RB-PDAT achieved complete resolution in 77% of severe progressive IK patients. Interestingly, in patients who went on to require TPK after RB-PDAT due to continued worsening of IK, graft survival at 1 year postoperatively was 86%, similar to published literature for graft survival in optical penetrating keratoplasty (OPK) [18]. While these clinical study results for RB-PDAT are promising, no RCT has been published. The Rose Bengal Electromagnetic Activation with Green Light for Infection Reduction (REAGIR) trial is an RCT that recently completed patient enrollment and will randomize moderate-to-severe vision loss (20/40 or worse) IK patients to adjuvant sham or RB-PDAT with standard-of-care topical antimicrobial therapy consisting of topical chlorhexidine 0.02% (acanthamoeba), natamycin 5% (fungus), or moxifloxacin 0.5% (unknown) with one year follow-up (ClinicalTrials.gov Identifier: NCT05110001).

## 4. Discussion

In conclusion, the diagnosis and management of IK remains a significant challenge for providers. Implementation of comprehensive clinical algorithms for IK patients can reduce vision-threatening complications and reduce unnecessary care [47]. Gold-standard corneal cultures have low positive culture rates. Hence, maximizing corneal culture positivity through drug holidays, minimizing contamination, and optimizing sample order are crucial [48]. Our culture sensitivity data for the top Gram-negative (*Pseudomonas aeruginosa*) and Gram-positive (*Staphylococcus aureus*) bacteria support the use of fortified vancomycin and tobramycin as first-line medical therapy for IK patients until patient-specific culture sensitivity data are available. Interestingly, the proportion of positive cultures at our institution that are bacterial has increased from 39% to 83.4% over the past decade, likely due to increased contact lens use [19]. MDS utility is currently limited by the need for specialized equipment, high financial cost (approximately USD 500 per swab), and long processing time (up to 7 days) [49]. Improving MDS technology will increase its access and applicability to IK patients. Image-based diagnosis of IK is an interesting research area, given that current gold-standard corneal cultures and MDS take both time and resources. A recent study found that a large international cohort of expert corneal specialists presented with a single corneal photo could correctly distinguish bacterial from fungal IK 72% of the time [50]. Artificial intelligence (AI) models represent a promising tool to improve corneal imaging-based diagnostic accuracy [51,52,53]. Increased pharmaceutical innovations in IK are needed but challenging given the lack of industry interest [54]. Thus, procedural advancements such as PACK-CXL and RB-PDAT fill an important clinical need. Results from RCTs, such as REAGIR, will provide valuable human clinical data to advance these therapies into clinical practice. Advances in genetic testing may allow for a more personalized medicine approach to IK management in the future. For example, single-nucleotide polymorphisms (SNPs) in host immune defense genes, including toll-like receptors (TLRs) and beta-defensin (DEFB1), can predispose individuals to IK [55]. Further research into novel antimicrobial medical and surgical treatments is needed to help reduce the high global burden of IK.

## Figures and Tables

**Table 1:** Bascom Palmer Eye Institute Corneal Culture Results (2011–2021).

	Isolates	Top Pathogen
**Bacteria**	**3010 (83.4%)**	
Gram-negative	1693 (46.9%)	Pseudomonas aeruginosa
Gram-positive	1231 (34.1%)	Staphylococcus aureus
Mycobacterium species	50 (1.4%)	Mycobacterium chelonae
Nocardia species	36 (1%)	Nocardia asteroides
**Fungus**	**486 (13.4%)**	
Mold	387 (10.7%)	Fusarium species
Yeast	99 (2.7%)	Candida albicans
**Parasite**	**113 (3.13%)**	
Acanthamoeba	113 (3.13%)	N/A

**Table 2:** Corticosteroid use for infectious keratitis.

Pathogen	Corticosteroid Summary
*BACTERIAL*	
Nocardia	Contraindicated (Srinivasan 2012) [33]
Mycobacteria	Contraindicated (Girgis 2012) [34]
Other	Improved outcomes for severe (BSCVA < 20/400, central, deep) bacterial corneal ulcers (Srinivasan 2012) [33] Early administration (<3 days) is better than late (Srinivasan 2012) [33]
*FUNGAL*	Contraindicated (Knutsson 2021) [35]
*PARASITIC*	
Acanthamoeba	Safe for treating ocular pain and discomfort after initiation of appropriate antiamoebic therapy (Carnt 2016) [28]
*VIRAL*	
Epithelial	Contraindicated in initial management (White 2014) [36]
Stromal	Use in conjunction with oral antiviral therapy (Wilhelmus 1994) [37]
Endothelial	Use in conjunction with oral antiviral therapy (White 2014) [36]
*UNKNOWN*	Contraindicated (Hirano 2021) [38]

BSCVA = best spectacle-corrected visual acuity.

**Table 3:** Recent clinical reports of photo-activated chromophore for keratitis corneal collagen cross-linking (PACK-CXL).

Author (Year)	Pathogen (n)	Study Type	Photosensitizer	Light Source (λ) Irradiance (Time) Fluence	Conclusions
Hafezi et al. (2022) [40]	Staphylococcus (3) Pseudomonas (3) Klebsiella, Serratia, Morganella, Escherichia (1) Mycobacteria (1) Aspergillus (2) Candida (1) Unknown (7)	Randomized clinical trial	Hypo-osmolar riboflavin 0.1%	UV-A (365 nm) 9 mW/cm^2^ (10 min or 13 min and 20 s) 5.4 J/cm^2^ or 7.2 J/cm^2^	Standard-of-care topical antimicrobial therapy vs. first-line standalone PACK-CXL for small (<4 mm) and partial-thickness (<350 μm) bacterial and fungal keratitis = Similar re-epithelialization times Overall success rate (complete resolution of signs of infection) for first-line standalone PACK-CXL = 88.9% (16/18)
Achiron et al. (2022) [44]	Pseudomonas (8) Staphylococcus (7) Streptococcus (4) Klebsiella (4) Moraxella (3) Serratia (2) Corynebacterium (1) Unknown (1)	Retrospective interventional cohort	Hypo-osmolar riboflavin 0.1%	UV-A (365 nm) 30 mW/cm^2^ (3 min) 5.4 J/cm^2^	Adjuvant PACK-CXL plus standard-of-care topical antimicrobial therapy = Better final CDVA, reduced re-epithelialization time, and less need for TPK compared to standard-of-care topical antimicrobial therapy alone for moderate (2–5 mm) to severe (>5 mm) partial-thickness (<300 μm) culture-positive bacterial keratitis
Prajna et al. (2021) [41]	Streptococcus (4) Pseudomonas (4) Moraxella (2) Nocardia (2) Other (5)	Randomized clinical trial	Iso-osmolar riboflavin 0.1%, 20% dextran	UV-A (365 nm) 3 mW/cm^2^ (30 min) 5.4 J/cm^2^	No benefit of adjuvant PACK-CXL in addition to standard-of-care topical Moxifloxacin 0.5% for bacterial keratitis
Gulias-Cañizo et al. (2020) [14]	Staphylococcus (14) Klebsiella (4) Serratia (2) Enterobacter (2) Bacillus (2) Fusarium (3) Mixed (6) Unknown (9)	Prospective observational cohort	Hypo-osmolar riboflavin 0.1%	UV-A (370 nm) 3 mW/cm^2^ (30 min) 5.4 J/cm^2^	Treatment-resistant IK (increased epithelial defect after one week of topical antimicrobial therapy) treated with PACK-CXL healed in 90.5% of cases by 3 months None of the 3 treatment-resistant fungal keratitis improved with PACK-CXL, necessitating surgery
Ozbek-Uzman et al. (2020) [45]	Streptococcus (3) Staphylococcus (2) Enterococcus (2) Klebsiella (1) Escherichia (1) Pseudomonas (1) Stenotrophomonas (1) Candida (5) Aspergillus (1) Fonsecaea (1) Unknown (4)	Retrospective interventional cohort	Iso-osmolar riboflavin 0.1%, 1.1% HPMC (CCT > 400 μm) Hypo-osmolar riboflavin 0.1% (CCT 350–400 μm)	UV-A (365 nm) 3 mW/cm^2^ (30 min) 5.4 J/cm^2^	Second-line PACK-CXL in post-PKP patients with topical antimicrobial resistant IK = Shorter complete healing time and higher ratio of patients with ≥2 Snellen lines of BCDVA No statistically significant difference in graft failure rates between PACK-CXL (27.8%) and non-CXL (54.5%)
Prajna et al. (2020) [13]	Fusarium (20) Aspergillus (7) Other: Bipolaris, Curvularias, Exserohilum, Scedosporium, Colletotrichum (11) Unidentified dematiaceous (5) Unknown (11)	Randomized clinical trial	Iso-osmolar riboflavin 0.1%, 20% dextran	UV-A (365 nm) 3 mW/cm^2^ (30 min) 5.4 J/cm^2^	No benefit of adjuvant PACK-CXL in addition to topical Natamycin or Amphotericin in the treatment of filamentous fungal keratitis (no improvement in microbiological cure, infiltrate and/or scar size, epithelialization, and risk of corneal perforation with need for TPK

**Table 4:** Clinical reports of Rose Bengal photodynamic antimicrobial therapy (RB-PDAT).

Author (Year)	Pathogen (n)	Study Type	Photosensitizer	Fluence	Conclusions
Sepulveda-Beltran et al. (2022) [18] and Naranjo et al. (2019) [16]	Acanthamoeba (16) Fusarium (4) Curvularia (2) Pseudomonas (2) Serratia (1) Proteus (1) Mycobacterium (1) Multiple (1) Unknown (3)	Retrospective interventional cohort	0.1% OR 0.2% Rose Bengal	5.4 J/cm^2^ (15 min)	Adjuvant RB-PDAT with topical antimicrobial therapy achieved complete clinical resolution (no need for TPK) in 77% of severe progressive IK patients In 23% of patients requiring TPK, graft survival at 1 year postoperatively was 86%
Bagga et al. (2022) [17]	Aspergillus (3) Fusarium (3) Acremonium (1)	Prospective case series	0.1% Rose Bengal	5.4 J/cm^2^ (15 min)	Adjuvant RB-PDAT with topical natamycin 5% every hour and oral ketoconazole 200 mg twice a day achieved complete clinical resolution (re-epithelialization and infiltrate resolution) in all 3 Fusarium patients, whereas all 3 Aspergillus patients and the 1 Acremonium patient clinically worsened, requiring TPK
Levine et al. (2021) [46]	Serratia (1)	Case report	0.1% Rose Bengal	5.4 J/cm^2^ (15 min)	Adjuvant RB-PDAT with topical antibacterial therapy halted stromal necrosis and achieved re-epithelialization in a case of severe progressive Serratia keratitis
Amescua et al. (2017) [15]	Fusarium (1)	Case report	0.1% Rose Bengal	0.9 J/cm^2^ (#1) and 1.8 J/cm^2^ (#2)	Adjuvant RB-PDAT × 2 sessions with topical, intrastromal, and oral antifungal therapy eradicated infection and prevented need for TPK in multidrug-resistant severe progressive Fusarium keratitis

# refers to 2 different tested fluences.
